# Pre-Existing Cross-Reactive Antibody Responses Do Not Significantly Impact Inactivated COVID-19 Vaccine-Induced Neutralization

**DOI:** 10.3389/fimmu.2021.772511

**Published:** 2021-11-19

**Authors:** Jin Wang, Cheng Guo, Lin Cai, Conghui Liao, Huaimin Yi, Qianlin Li, Huan Hu, Qiang Deng, Yuying Lu, Zhongmin Guo, Zeliang Chen, Jiahai Lu

**Affiliations:** ^1^ Department of Epidemiology, School of Public Health, Sun Yat-sen University, Guangzhou, China; ^2^ National Medical Products Administration (NMPA) Key Laboratory for Quality Monitoring and Evaluation of Vaccines and Biological Products, Guangzhou, China; ^3^ Center for Infection and Immunity, Mailman School of Public Health, Columbia University, New York, NY, United States; ^4^ Futian District Center for Disease Control and Prevention, Shenzhen, China; ^5^ Laboratory Animal Center, Sun Yat-sen University, Guangzhou, China

**Keywords:** SARS-CoV-2, seasonal coronavirus, inactivated vaccine, neutralization, cross-reactive antibody immunity

## Abstract

Recent exposure to seasonal coronaviruses (sCoVs) may stimulate cross-reactive antibody responses against severe acute respiratory syndrome CoV 2 (SARS-CoV-2). However, previous studies have produced divergent results regarding protective or damaging immunity induced by prior sCoV exposure. It remains unknown whether pre-existing humoral immunity plays a role in vaccine-induced neutralization and antibody responses. In this study, we collected 36 paired sera samples from 36 healthy volunteers before and after immunization with inactivated whole-virion SARS-CoV-2 vaccines for COVID-19, and analyzed the distribution and intensity of pre-existing antibody responses at the epitope level pre-vaccination as well as the relationship between pre-existing sCoV immunity and vaccine-induced neutralization. We observed large amounts of pre-existing cross-reactive antibodies in the conserved regions among sCoVs, especially the S2 subunit. Excep t for a few peptides, the IgG and IgM fluorescence intensities against S, M and N peptides did not differ significantly between pre-vaccination and post-vaccination sera of vaccinees who developed a neutralization inhibition rate (%inhibition) <40 and %inhibition ≥40 after two doses of the COVID-19 vaccine. Participants with strong and weak pre-existing cross-reactive antibodies (strong pre-CRA; weak pre-CRA) had similar %inhibition pre-vaccination (10.9% ± 2.9% *vs.* 12.0% ± 2.2%, *P*=0.990) and post-vaccination (43.8% ± 25.1% *vs.* 44.6% ± 21.5%, *P*=0.997). Overall, the strong pre-CRA group did not show a significantly greater increase in antibody responses to the S protein linear peptides post-vaccination compared with the weak pre-CRA group. Therefore, we found no evidence for a significant impact of pre-existing antibody responses on inactivated vaccine-induced neutralization and antibody responses. Our research provides an important basis for inactivated SARS-CoV-2 vaccine use in the context of high sCoV seroprevalence.

## Introduction

Coronavirus disease 2019 (COVID-19), caused by severe acute respiratory syndrome coronavirus 2 (SARS-CoV-2) infection, has led to widespread human and economic losses. The SARS-CoV-2 belongs to the Coronaviridae family and the Coronavirinae subfamily, which are divided into four genera (α-, β-, γ-, and δ-CoVs). Both α and β CoVs mainly infect mammals, among which four seasonal CoVs (sCoVs), including αCoVs (NL63 and 229E) and β CoVs (OC43 and HKU1), are endemic globally and account for 10%–30% of upper respiratory tract infections (1). SARS-CoV-2 has four important structural proteins, including spike (S), envelope (E), membrane (M) and nucleocapsid (N) proteins. SARS-CoV-2 is thought to induce antigen-specific antibody responses through S and N proteins. The S glycosylated protein contains the receptor-binding domain (RBD) that is responsible for recognizing and binding to the host cell receptor angiotensin-converting enzyme 2 (ACE2) (2). Antibodies targeting the RBD have been suggested to account for >90% of neutralizing activity in SARS-CoV-2 convalescent sera (3). SARS-CoV-2 shares a 79.7% nucleotide identity with the sequence of SARS-CoV, having 90% homology in N protein and 76% homology in S protein (4). In addition, SARS-CoV-2 S protein has 23%–30% homology with four sCoVs (5). Because sCoVs are common and infect people repeatedly during their lives, the seroprevalence in adults in some regions can be more than 80%–90% for NL63, 229E and OC43, which is much higher than the rates in children (6, 7). However, antibody titers induced by sCoVs are short-lived and fluctuate over time (8).

*In vitro* peptide stimulation and crystal structure analysis showed T cell-mediated cross-recognition to circulating β CoV (OC43 and HKU1) (9). Additionally, T cell-mediated anti-SARS-CoV-2 responses were found in unexposed human, indicating a cross-reaction between human CoVs and SARS-CoV-2 (10–14). Accumulating evidence suggests that pre-existing humoral immunity to sCoVs may play a role in the specific anti-SARS-CoV-2 antibody responses (15), but this possibility is controversial (16, 17). Some studies showed that elevated levels of pre-existing antibodies against sCoVs, specifically OC43 and HKU1, were associated with a less severe course of COVID-19, suggesting a protective effect of prior exposure to sCoVs (18–20). In contrast, laboratory evidence indicates possible antibody-dependent enhancement (ADE) of SARS-CoV-2 infection by previous sCoV immunity (21, 22). Concerns about the ADE or original antigenic sin (OAS) effect induced by pre-existing antibody responses mainly come from the past evidence in dengue virus and influenza virus (23, 24). Although several studies have been carried out, there is currently no conclusive evidence that pre-existing immune responses to sCoVs induce neutralization or enhancement of SARS-CoV-2 antibodies. During the current outbreak, COVID-19 vaccination is a critical prevention measure to help end the COVID-19 pandemic by achieving sufficient population immunity. As COVID-19 vaccines have been administered across the world, it is necessary to understand the factors that affect the effectiveness or safety of these vaccines. This exacerbates the urgent need for laboratory data to determine the relationship between pre-existing immunity to sCoVs and the effectiveness of COVID-19 vaccines against SARS-CoV-2.

In this study, we collected paired serum samples from 36 healthy volunteers before and after immunization with inactivated whole-virion SARS-CoV-2 vaccines and analyzed the distribution and intensity of pre-existing antibody responses at the epitope level pre-vaccination as well as the relationship between pre-existing sCoV immunity and vaccine-induced neutralization.

## Methods and Materials

### Serum Specimens

Serum specimens were collected from 36 healthy volunteers who received inactivated SARS-CoV-2 vaccines (Sinopharm, China) between 16 December, 2020 and 10 February, 2021. Before vaccination, all volunteers gave informed consent for participation. All participants reported not having a prior history of COVID-19 disease. All participants were negative both for SARS-CoV-2 nucleic acid on nasopharyngeal swab specimens by reverse transcriptase polymerase chain reaction (RT-PCR) assays and specific IgG/IgM antibody tests of serum samples by commercial enzyme-linked immunosorbent assay (ELISA) kits (BGI PathoGenesis Pharmaceutical Tech, China). The volunteers received two doses of inactivated SARS-CoV-2 vaccine with a 28–day interval. Serum specimens were collected before vaccination (n=36) and 2–4 weeks after the second injection (n=36) from the same volunteers by healthcare professionals of the Futian District Center for Disease Control and Prevention, Shenzhen, China.

### Microarray Fabrication

**Figure 1:**
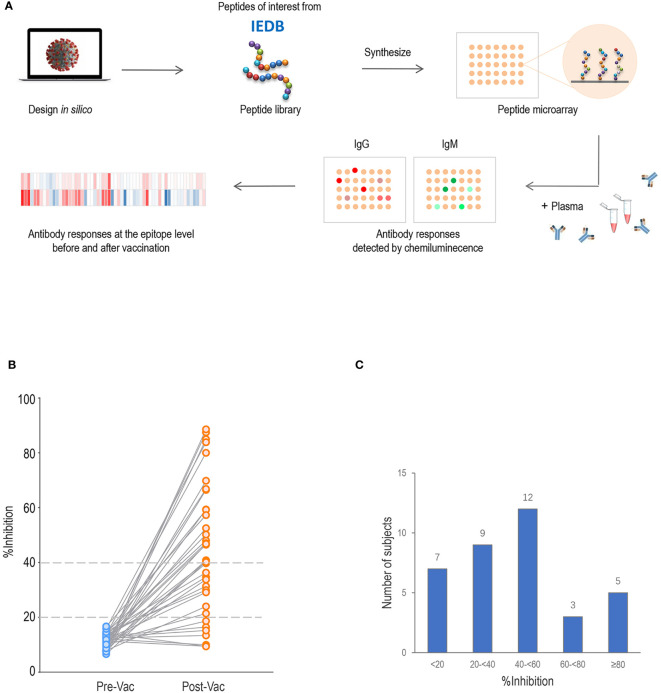
Proteome microarray and neutralization inhibition rate (%inhibition) of serum samples. **(A)** Schematic illustration of SARS-CoV-2 proteome microarray fabrication and application. **(B)** The %inhibition of pre-vaccination (Pre-Vac) and post-vaccination (Post-Vac) sera samples from 36 healthy volunteers vaccinated with inactivated COVID-19 vaccines. **(C)** The number of participants with %inhibition <20%, 20% to <40%, 40% to <60%, 60–<80% and ≥80%.

The procedure for microarray fabrication is illustrated in **Figure 1A**. The epitope information for SARS-CoV-2 was collected from the Immune Epitope Database (IEDB; http://www.iedb.org/) in January 2021. The sequences were clustered using the Epitope Cluster Analysis tool (http://tools.iedb.org/) with a minimum sequence identity of 70%. Finally, a peptide library, which contained 243 peptides representing SARS-CoV-2, was prepared. The synthetic peptides were designed to cover almost the entire S (coverage 97.6%), N (coverage 95.0%) and M (coverage 94.2%) proteins. The peptide library was based on the proteome sequence of the virus isolate Wuhan-Hu-1, and each peptide in the library ranged from 9–30 amino acids in length. The peptides were synthesized by GL Biochem (Shanghai, China).

The peptides at random positions on the array, along with negative and positive controls (anti-human IgG and IgM), were printed in duplicate on slides to generate identical arrays in a 2×8 subarray format using a printer (Scienion AG, Monmouth Junction, NJ, USA). The microarrays were stored at −80°C until use.

### Microarray-Based Serum Detection

The microarrays stored at −80°C were warmed to room temperature and then incubated in blocking buffer for 1 h. A total of 100 μL diluted serum (1:100) or dilution buffer only added for incubation with each subarray overnight at 4°C. The arrays were washed, and bound antibodies were detected after incubation with Cy3-conjugated goat anti-human IgG and Cy5 AffiniPure donkey anti-human IgM (Jackson ImmunoResearch, PA, USA) at room temperature for 1–2 h. After washing, the microarrays were scanned with an InnoScan 300 Microarray Scanner (Innopsys, Chicago, IL USA) at 10-μm resolution using lasers at 532 nm for IgG and 640 nm for IgM. Fluorescence signal intensities with background subtraction were extracted using GenePix Pro7 software (Molecular Devices, San Jose, CA, USA).

### Surrogate Neutralization ELISA

Circulating neutralizing antibodies against SARS-CoV-2 in sera were detected using an Anti-SARS-CoV-2 Neutralizing Antibody ELISA Kit (Vazyme Biotech, China), with positive (human SARS-CoV-2 neutralizing antibody) and negative controls. The microtiter plate was pre-coated with ACE2, and samples were added to the appropriate microtiter plate wells with the horseradish peroxidase (HRP)-conjugated RBD of the SARS-CoV-2 spike glycoprotein. A competitive inhibition reaction occurred between the HRP-conjugated RBD protein and SARS-CoV-2 neutralizing antibody in samples. After thorough washing, a substrate solution was added to the wells and incubated for 15 min. The enzyme reaction was stopped, and the color intensity was measured spectrophotometrically at 450 nm. Color development was inversely proportional to the amount of SARS-CoV-2 neutralizing antibody in the sample. The optical density (OD) values were corrected by subtracting blank readings from sample or control readings. Neutralizing activity was determined by calculating the percent inhibition of the SARS-CoV-2 spike RBD–ACE2 interaction (%inhibition). The %inhibition was calculated by the formula: (1– average OD value of the serum sample/average OD value of negative controls) ×100%. According to the manufacturer’s instructions, an inhibition rate **≥**20% was considered as positive, indicating the presence of SARS-CoV-2 neutralizing antibody. A %inhibition **≥**40% was considered indicative of stronger neutralizing activity.

### Data Analysis

The background-corrected fluorescence intensities for IgG and IgM response values were used for analysis. Normalization among the microarray slides was performed. The signal intensity for each peptide was determined from the average fluorescence signal of replicate spots. The individuals with a stronger pre-existing antibody response were defined as those having more than five reactive peptides, against which IgG signal intensities exceeded mean + 2 standard deviation (SD) readings. Protein sequences for SARS-CoV-2 and four sCoVs (NL63, 229E, OC43 and HKU1) were downloaded from the US National Center for Biotechnology Information (NCBI) database. Multiple sequence alignments for four sCoVs and SARS-CoV-2 were performed using Geneious Prime 2019.

Statistical analyses were performed using SPSS statistics 23 (SPSS, Inc., Armonk, NY, USA) and GraphPad 7 (GraphPad Software, San Diego, CA, USA). Patient demographic data are expressed as mean ± SD or as numbers (percentages). Data plotted in logarithmic scales are expressed as median (25^th^ percentile, 75^th^ percentile). Comparisons of data between groups were performed using independent samples t test or nonparametric Mann–Whitney U test as appropriate. Categorical variables were analyzed using the Chi-Square test and Fisher’s exact test. A two-tailed *P*<0.05 was considered statistically significant.

## Results

### Participants and Sample Collection

We examined the SARS-CoV-2 epitope-specific antibody responses in 36 individuals who were vaccinated with two doses of the Sinopharm inactivated COVID-19 vaccine (interval 28.2 ± 0.9 days). The participants had a mean age of 36.8 ± 9.8 years (range, 24–57 years). Paired blood samples were collected before vaccination and 19.0 ± 3.6 days after the second vaccination.

All 36 pre-vaccination sera samples tested negative for neutralizing activity (%inhibition <20; **Figure 1B**). By 2–4 weeks after the second subcutaneous injection of inactivated vaccine, 80.6% (n=29) of participants had developed a %inhibition **≥**20, and 55.6% (n=20) had developed a %inhibition **≥**40. The numbers of participants with %inhibition <20%, 20% to <40%, 40% to <60%, 60% to <80%, and **≥**80% were 7, 9, 12, 3 and 5 after vaccination, respectively (**Figure 1C**). For further analysis, comparisons were made between two groups with %inhibition <40 (n=16, 44.4%) and %inhibition **≥**40 (n=20, 55.6%). No differences in age, gender distribution, vaccine strain, time interval between the first and second doses, and time of sample collection were observed between the two groups (all *P*>0.05; **Table 1**).

**Table 1:** Basic characteristics of the study participants.

	Overall (n=36)	%inhibition<40 (n=16)	%inhibition ≥40 (n=20)	*P* values
Age, years	36.8 ± 9.8	38.5 ± 11.0	35.4 ± 8.7	0.357
Male, n (%)	19 (52.8%)	9 (56.3%)	10 (50%)	0.749
Vaccine strains				0.315
WIV04, n (%)	18 (50.0)	10 (62.5)	8 (40.0)	
HB02, n (%)	18 (50.0)	6 (37.5)	12 (60.0)	
Time interval between the two vaccine doses	28.2 ± 0.9	28.1 ± 1.0	28.3 ± 0.8	0.434
Time interval between 2^nd^ vaccine dose and 2^nd^ sample collection	19.0 ± 3.6	19.2 ± 3.6	18.9 ± 3.6	0.782

### Impact of Pre-Existing Cross-Reactive Antibodies on COVID-19 Vaccine-Induced Neutralization

**Figure 2:**
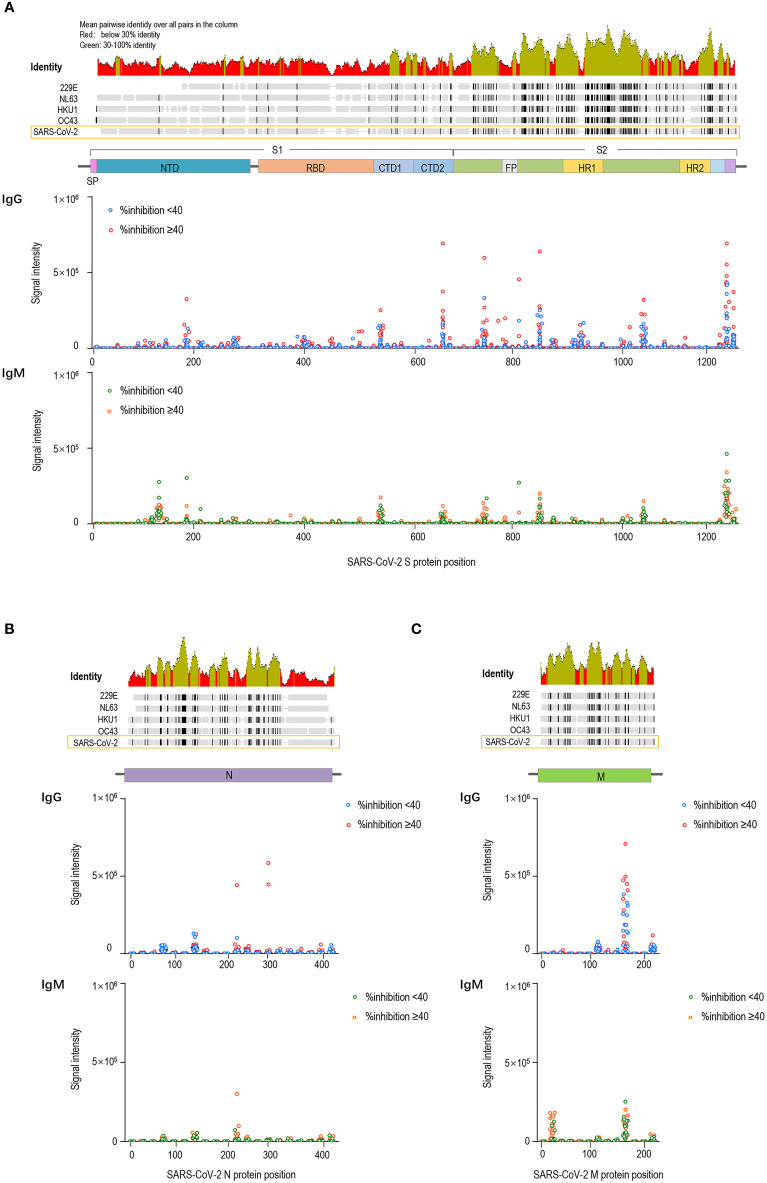
Pre-existing cross-reactive antibodies to SARS-CoV-2 in pre-vaccination sera. The dots represent pre-existing antibody responses in pre-vaccination sera from the vaccinees who acquired %inhibition <40 and %inhibition≥40 after two doses of the COVID-19 vaccine. The signal intensities of each peptide against IgG and IgM were separately mapped to the corresponding S **(A)**, M **(B)** and N **(C)** protein sequences.

We compared the pre-existing antibody responses in pre-vaccination sera from vaccinees who acquired %inhibition <40 and %inhibition **≥**40 after two doses of the COVID-19 vaccine. The signal intensities for each peptide against IgG and IgM antibodies were separately mapped to the corresponding S, M and N protein sequences. Moreover, the full-length S, M and N sequences of SARS-CoV-2 and sCoVs (NL63, 229E, OC43 and HKU1) were aligned. We found considerable antibody reactivities against SARS-CoV-2 in the pre-vaccination sera (**Figure 2**). The main targeted regions were largely shared between the IgG and IgM antibodies. Moreover, cross-reactive binding to the S protein was concentrated on highly conserved regions of hCoVs, especially the S2 subunit, which is consistent with higher degrees of homology in the S2 subunit than in the S1 among human CoVs (25, 26). Based on prior literature, it is speculated that these cross-reactive antibody responses might be generated by prior exposure to human CoVs (27). To facilitate the analysis, the signal intensities were logarithmically processed and compared. No significant differences in IgG antibody fluorescence intensities against S, M and N peptides were detected between the %inhibition <40 and %inhibition **≥**40 groups. Consistently, pre-existing IgM signal intensities were similar at S, M and N peptides between the two groups, except for IgM antibodies against S614–S633, M11–28 and N237–260, which were stronger in the %inhibition **≥**40 group. The comparisons of pre-existing cross-reactive antibody responses against S, M and N linear peptides between the two groups are presented in **Supplementary Table 1**. These data showed that except for a few peptides, the pre-existing IgG and IgM antibody responses were similar among vaccinated participants who acquired high and low neutralization inhibition rates after vaccination.

**Figure 3:**
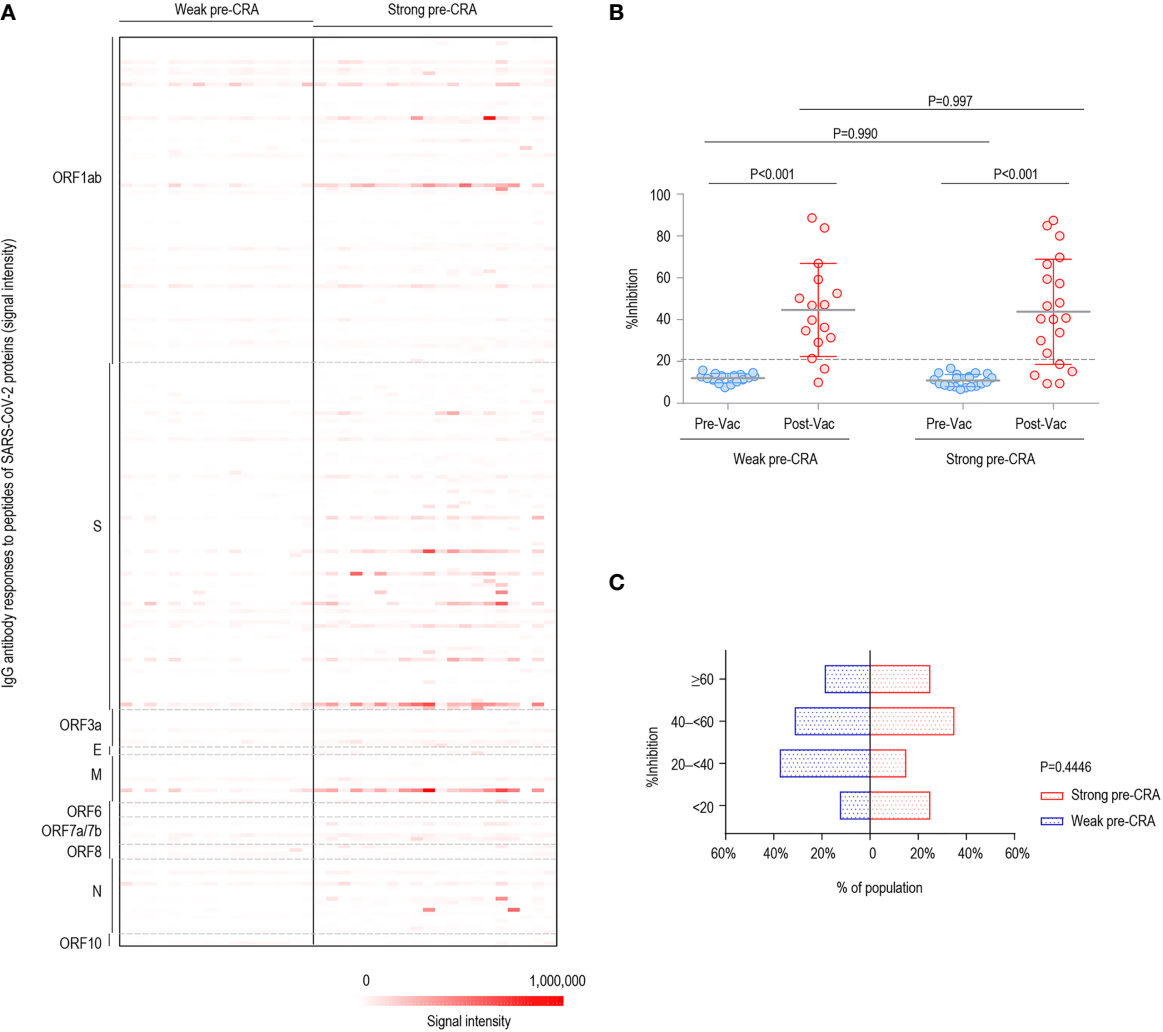
The impact of pre-existing cross-reactive antibodies on inactivated COVID-19 vaccine-induced neutralization. The participants were divided into two groups with strong and weak pre-existing cross-reactive antibodies (Strong pre-CRA, n=20; Weak pre-CRA, n=16). The individuals with a stronger pre-existing antibody response were defined as those having more than five reactive peptides, against which IgG signal intensities exceeded mean + 2 SD readings. **(A)** The strong pre-CRA group had stronger fluorescence intensities, indicating higher levels of pre-existing antibodies. **(B)** The %inhibition between the strong pre-CRA and weak pre-CRA groups. **(C)** The proportions of participants with %inhibition <20%, 20% to <40%, 40% to <60% and ≥60% in the strong pre-CRA and weak pre-CRA groups. Pre-Vac, pre-vaccination; Post-Vac, post-vaccination.

Subsequently, the participants with strong and weak pre-existing cross-reactive antibodies were divided into two groups (strong pre-CRA, n=20; weak pre-CRA, n=16). It can be seen from **Figure 3A** that the strong pre-CRA group had stronger fluorescence intensities, indicating higher levels of pre-existing antibodies. When we compared the %inhibition between the two groups, the %inhibition before vaccine immunization did not differ significantly between the strong pre-CRA and weak pre-CRA groups (10.9% ± 2.9% *vs.* 12.0% ± 2.2%, *P*=0.990). Again, after vaccine immunization, the %inhibition ​​did not differ between the strong pre-CRA and weak pre-CRA groups (43.8% ± 25.1% *vs.* 44.6% ± 21.5%, respectively, *P*=0.997; **Figure 3B**). Also, the proportions of participants with %inhibition <20%, 20% to <40%, 40% to <60% and ≥60% in the strong pre-CRA group were 25.0%, 15.0%, 35.0%, and 25.0% after vaccination, respectively; while the corresponding proportions in the weak pre-CRA group were 12.5%, 37.5%, 31.3% and 18.8%, respectively (**Figure 3C**). No significant differences in these proportions were observed between the two groups (*P*=0.4446). These data indicate that pre-existing cross-reactive antibodies do not have a significant impact on inactivated vaccine-induced neutralization.

### Impact of Pre-Existing Cross-Reactive Antibodies on Vaccine-Induced IgG and IgM Responses

The main viral target of interest for vaccines against SARS-CoV-2 is the S protein, which decorates the surface of the virus and is involved in receptor recognition, viral attachment, and entry into host cells (28). Thus, we focused on the changes in antibody responses to the S protein of SARS-CoV-2. We analyzed both IgG and IgM antibody responses to linear peptides of the S protein after vaccination between the groups with %inhibition <40 and %inhibition **≥**40. The results showed that IgG responses to six peptides, S544–573, S564–583, S574–603, S776–800, S1106–1125 and S1136–1165, exhibited significantly higher fluorescence intensities in the group with %inhibition ≥40. Similarly, the fluorescence intensities of IgM to six peptides, including S203–226, S544–573, S574–603, S756–780, S811–835 and S1106–1125, were higher in the group with %inhibition **≥**40, and the differences achieved statistical significance (**Supplementary Table 2**). The significant epitopes clustered in three regions, a CTD in the S1 subunit (aa544–603), the fusion peptide and the S2 cleavage site (aa756–835), and the “stem helix” around the HR2 in the S2 subunit (aa1106–1165), which were consistent with the previously identified significant epitopes of SARS-CoV-2 (29, 30).

**Figure 4:**
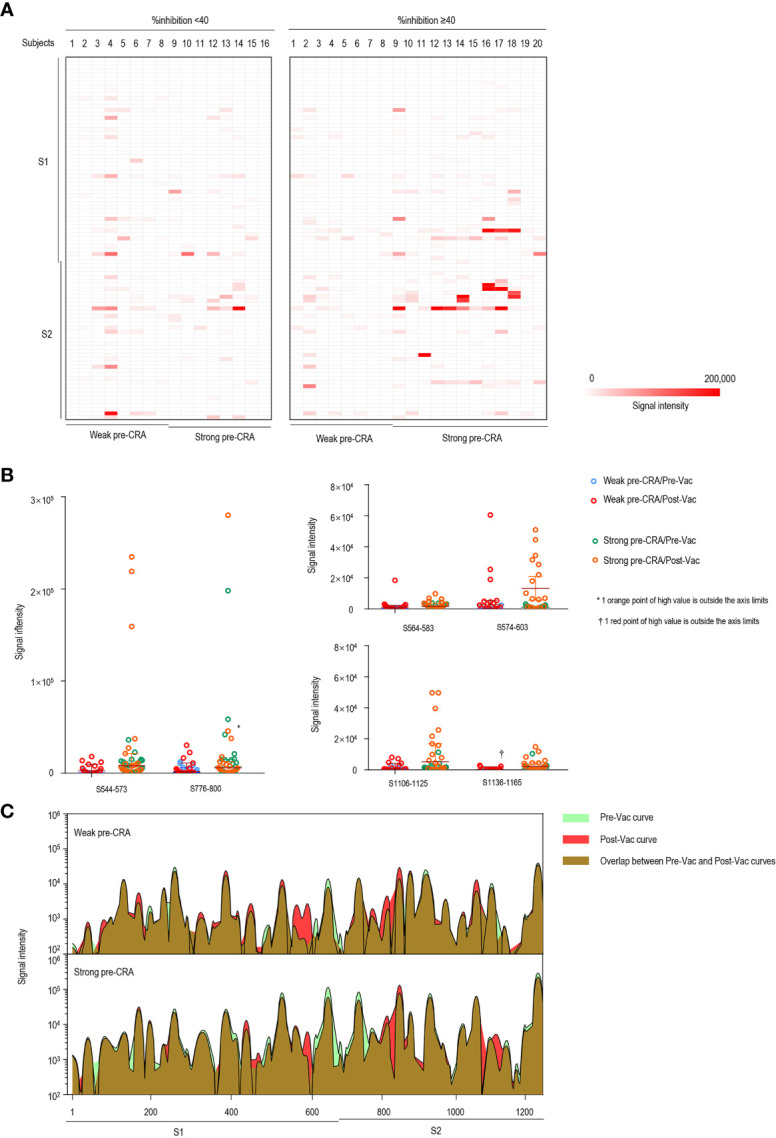
The impact of pre-existing cross-reactive antibodies on vaccine-induced IgG response. **(A)** The IgG antibody responses to linear peptides of the S protein in vaccinated individuals. The heatmap is formed by subtracting the corresponding pre-vaccination fluorescence readings from post-vaccination readings, representing the increase in fluorescence intensity after immunization. **(B)** The changes in IgG signal intensities to six significant epitopes in paired sera before and after vaccine immunization, as separately shown in the strong pre-CRA and weak pre-CRA groups. **(C)** Global profiles of IgG responses to linear peptides of the S proteins in the strong pre-CRA (n=20) and weak pre-CRA (n=16) sera. The graphs are plotted as median values of the IgG responses to linear peptides of the S protein. Blue, pre-vaccination curve; Red, post-vaccination curve; Purple, the overlap between pre-vaccination and post-vaccination curves. Pre-Vac, pre-vaccination; Post-Vac, post-vaccination.

**Figure 5:**
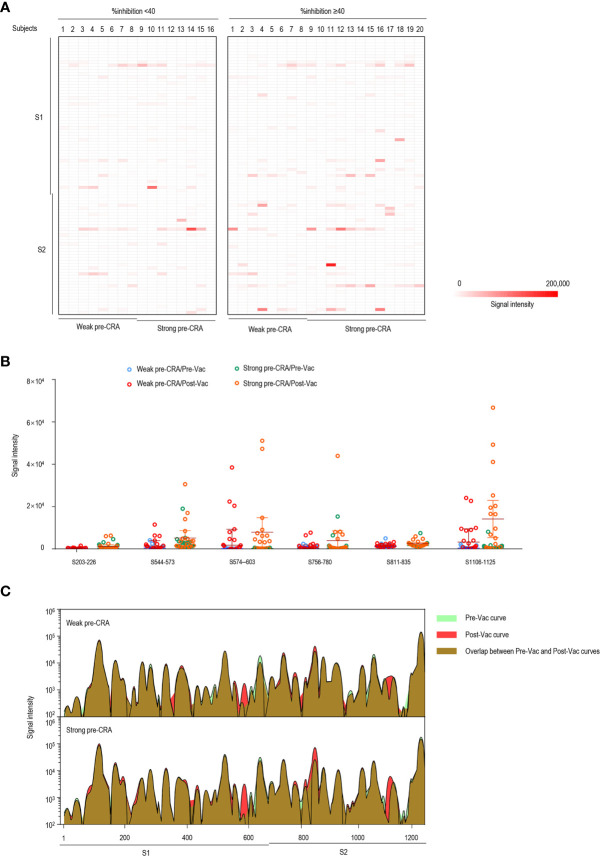
The impact of pre-existing cross-reactive antibodies on vaccine-induced IgM response. **(A)** The IgM antibody responses to linear peptides of the S protein in vaccinated individuals. The heatmap is formed by subtracting the corresponding pre-vaccination fluorescence readings from post-vaccination readings, representing the increase in fluorescence intensity after immunization. **(B)** The changes in IgM signal intensities to six significant epitopes in paired sera before and after vaccine immunization, as separately shown in the strong pre-CRA and weak pre-CRA groups. **(C)** Global profiles of IgM responses to linear peptides of the S proteins in the strong pre-CRA (n=20) and weak pre-CRA (n=16) sera. The graphs are plotted as median values of the IgM responses to linear peptides of the S protein. Blue, pre-vaccination curve; Red, post-vaccination curve; Purple, the overlap between pre-vaccination and post-vaccination curves. Pre-Vac, pre-vaccination; Post-Vac, post-vaccination.

We present the epitope-binding IgG/IgM antibody responses in sera of the two groups after subtracting the corresponding pre-vaccination signal intensities, which represents the increase in fluorescence intensity after immunization, as displayed in **Figure 4A** (IgG) and **Figure 5A** (IgM). Generally, the strength of reactive fluorescence was greater for IgG antibody than for IgM antibody, although the overall patterns of reactivity were similar. This is probably due to a rapid decline of IgM responses while IgG responses are more stable or wane at a slower rate. As shown in **Figure 4A**, we observed a trend towards higher responses in the strong pre-CRA group for some of the epitopes. Thus, IgG/IgM responses to the significant epitopes were compared between the strong pre-CRA and weak pre-CRA groups. For IgG, the strong pre-CRA group was more likely to produce a higher antibody response at some of the epitopes, including S574–603 and S1106–1125 (**Figure 4B**). At certain epitopes, such as S544–573 and S776–800, a few participants in the strong pre-CRA group exhibited extremely high values. These data suggest that strong responses against SARS-CoV-2 in a COVID-19 vaccinated population with pre-existing cross-reactive antibody are highly individual specific. A consistent trend was also observed for IgM responses (**Figure 5B**).

We next compared the overall antibody responses to S linear peptides between the strong pre-CRA and weak pre-CRA groups. We graphed the median values of IgG (**Figure 4C**) and IgM responses (**Figure 5C**) to linear peptides of the S protein. The strong pre-CRA and weak pre-CRA groups displayed similar patterns in response to IgG or IgM antibodies. For the significant epitopes mentioned above, the changes in fluorescence intensities from before to after vaccine immunization did not differ significantly between the strong pre-CRA and weak pre-CRA groups (*P*=0.390, *P*=0.610, *P*=0.702, *P*=0.214, *P*=0.265 and *P*=0.464 for S544–573, S564–583, S574–603, S776–800, S1106–1125 and S1136–1165 responses to IgG antibodies, respectively; and *P*=0.279, *P*=0.679, *P*=0.588, *P*=0.774, *P*=0.504 and *P*=0.656 for S203–226, S544–573, S574–603, S756–780, S811–835 and S1106–1125 responses to IgM antibodies, respectively). Overall, the strong pre-CRA group did not show a more significant increase in antibody responses to the S linear peptides from before to after vaccine immunization as compared with the weak pre-CRA group (**Figure 4C**, IgG; and **Figure 5C**, IgM).

## Discussion

In previous studies of pre-pandemic cohorts, 4.2%–4.9% of serum samples displayed IgG reactivity against the SARS-CoV-2 S antigen, indicating cross-reactivity between SARS-CoV-2 and sCoVs (7, 31). Immunity to sCoVs appears to last approximately 1 year, and re-infection with the same CoV frequently occurs beyond 12 months after infection (32). Repeated infection leads to a high seroprevalence of sCoVs, with studies showing seroprevalence rates of 82.9% for OC43, 82.1% for 229E, 74.6% for NL63, and 59.2% for HKU-1 (31). Due to the high prevalence, a major hurdle in estimating the seroprevalence of IgG against sCoVs is the lack of a true negative reference population. Thus, accurate detection of sCoV exposure is not easy. In this study, we did not distinguish the exact CoVs that induced pre-existing antibody responses; instead, we mapped epitope-binding antibody responses before and after vaccine immunization. We observed pre-existing antibody reactivity in the conserved regions among human CoVs, especially the S2 subunit, which is consistent with previous evidence that the S2 subunit is the predominant target of pre-existing SARS-CoV-2 spike protein cross-reactive antibodies (26). Speculation that the pre-existing antibody responses are mainly derived from prior sCoV exposure requires further exploration in future studies.

Recent efforts have been made to explore the impact of prior immunity to sCoVs on SARS-CoV-2 infection, neutralization and disease severity, and the results are divergent among studies. Some studies have shown that the pre-existing immune response to sCoVs mitigates the symptoms of SARS-CoV-2 infection and reduces the occurrence of severe COVID-19, with benefits including lower rates of intensive care unit (ICU) admission and death (33, 34). Galipeau et al. found a broad range of neutralizing activity (0–45%) in pre-pandemic serum samples, especially for NL63 and OC43 antibodies, which could interfere with the binding of SARS-CoV-2 to ACE2 receptors, so as to confer protection (31). In contrast, some studies have revealed that the highly cross-reactive OC43 S-IgG antibody titer, as a risk factor, is related to COVID-19 severity (16). Also, Focosi et al. showed an association between previous humoral immunity to NL63 and 229E and worse clinical outcome in COVID-19 patients (21). Other studies have suggested that pre-existing immunity to sCoVs may not be related to COVID-19 severity or protection at all (17, 27, 35). Currently, few studies have provided evidence for an impact of pre-existing cross-reactive antibodies on inactivated COVID-19 vaccine-induced neutralization.

In the present study, we found no evidence of cross-protection linked to pre-existing sCoV immune reactivity. First, there was no significant difference in the strength of pre-existing antibodies in pre-vaccination sera from individuals who developed high and low RBD neutralization, except for a few peptides. Second, the neutralization inhibition rates were similar in pre-vaccination sera, regardless of the presence of strong or weak pre-existing antibodies. These results are consistent with the data from Anderson et al. (7), who found that pre-pandemic serum samples had very low or undetectable levels of neutralizing antibodies by neutralization assays, regardless of the presence of cross-reactive antibodies against SARS-CoV-2 S or N. Third, the participants with strong and weak pre-existing antibodies showed similar neutralizing activity after vaccination. Therefore, no evidence indicates a link between pre-existing antibodies and inactivated vaccine-induced neutralization. Our data are inconsistent with some studies that have reported protective immunity induced by prior exposure to sCoVs (18–20). It is possible that pre-existing immunity to sCoVs may play a protective role through non-RBD neutralization or non-neutralizing effects. As we know, the RBD is a region rich in conformational epitopes but not linear epitopes (30) and is the target of >90% of neutralizing antibodies in COVID-19 cases (3). However, a few non-RBD neutralizing epitopes are also identified on the S1, S2 or even N protein (36). Whether pre-existing antibodies can affect SARS-CoV-2 infection or vaccine immunization through non-neutralizing effects or cellular immunity-mediated protective effects was not assessed in this study and requires further investigation.

We compared the IgG and IgM responses to the S protein linear peptides after vaccine immunization in the groups with %inhibition <40 and %inhibition ≥40. After vaccination, IgG or IgM antibody responses to several linear peptides were significantly higher in individuals with %inhibition **≥**40. These epitopes are mainly located in three regions of the S protein, which is consistent with the identified SARS-CoV-2 epitopes reported in previous studies (29, 30). We found that the strong pre-CRA group was more likely to produce higher antibody responses at some of the epitopes, such as S574–603 and S1106–1125. Consistent with previous evidence, vaccinees who were previously infected with SARS-CoV or SARS-CoV-2 had a significantly higher antibody response than previously uninfected vaccinees (37–42). However, upon analysis of the global profiles of antibody responses to the S protein peptides, the strong pre-CRA and weak pre-CRA groups showed similar patterns in response to IgG or IgM antibodies. These results may indicate that only a small group of people with pre-existing antibodies may develop additionally increased antibody responses to inactivated COVID-19 vaccines; overall though, the pre-existing antibodies do not further significantly increase the antibody response after vaccination. The difference in vaccine-induced immune responses among people with pre-existing antibodies may be due to individual differences or varied sources of pre-existing antibodies, such as αCoVs (NL63 and 229E) and β CoVs (OC43 and HKU1). It should be noted that we employed a method based on binding to linear peptides to characterize the cross-reactivity of sera antibodies, which did not take into consideration the steric or 3D conformation of the whole SARS-CoV-2 proteins. Therefore, this method may not accurately capture antibodies that bind to viral proteins and have *in vivo* relevance, which is a limitation of our study. These data preliminarily reflect the changes of IgG and IgM responses to the S linear peptides before and after inactivated vaccine immunization in sera with different levels of pre-existing antibodies. Confirmation of these findings will require further investigation *via* quantitative research with a larger sample size.

In conclusion, through peptide microarray-based analysis, we found a considerable amount of pre-existing antibody responses to SARS-CoV-2 S, M and N proteins. However, our study produced no significant evidence that pre-existing antibody responses have an impact on inactivated COVID-19 vaccine-induced neutralization. Our research provides an important basis for use of inactivated COVID-19 vaccines in the context of high sCoV seroprevalence. However, further studies are needed to deeply understand the relationship between prior sCoV exposure and COVID-19 vaccine effectiveness, with features such as longitudinal cohorts and experimental neutralization assays and ideally assessing both humoral and cellular immunity.

## Data Availability Statement

The datasets presented in this study can be found in online repositories. The names of the repository/repositories and accession number(s) can be found below: ArrayExpress; E-MTAB-11084 and E-MTAB-11117.

## Ethics Statement

The studies involving human participants were reviewed and approved by The Ethics Committee of the School of Public health, Sun Yat-sen University. The participants provided their written informed consent to participate in this study.

## Author Contributions

JL, ZC, and ZG developed the conceptual ideas and designed the study. JW, CG, CL, HY, QL, HH, and QD performed experiments. LC collected research specimens. JW, CG, ZG, and YL performed data analysis. JW wrote the manuscript and prepared figures with suggestions from other authors. JL, ZC, and CG revised the manuscript. All authors contributed to the article and approved the submitted version.

## Funding

This work was funded by the National Key Research and Development Project (2018YFE0208000) and the Key-Area Research and Development Program of Guangdong Province (2018B020241002).

## Conflict of Interest

The authors declare that the research was conducted in the absence of any commercial or financial relationships that could be construed as a potential conflict of interest.

## Publisher’s Note

All claims expressed in this article are solely those of the authors and do not necessarily represent those of their affiliated organizations, or those of the publisher, the editors and the reviewers. Any product that may be evaluated in this article, or claim that may be made by its manufacturer, is not guaranteed or endorsed by the publisher.

## Supplementary Material

Supplementary Table 1 The pre-existing cross-reactive antibody responses (IgG and IgM) in pre-vaccination sera. Fluorescence readings are logarithmically processed.

Supplementary Table 2 The antibody responses (IgG and IgM) in post-vaccination sera. Fluorescence readings are logarithmically processed.
